# Supplementary material for: Primordial neon and the deep mantle origin of kimberlites
Source: Nat Commun. 2025 Apr 6;16:3281. doi: 10.1038/s41467-025-58625-5 (PMC11972409; doi:10.1038/s41467-025-58625-5)
Supplement: Supplementary file 1 — Supplementary Information [file 41467_2025_58625_MOESM1_ESM.pdf]

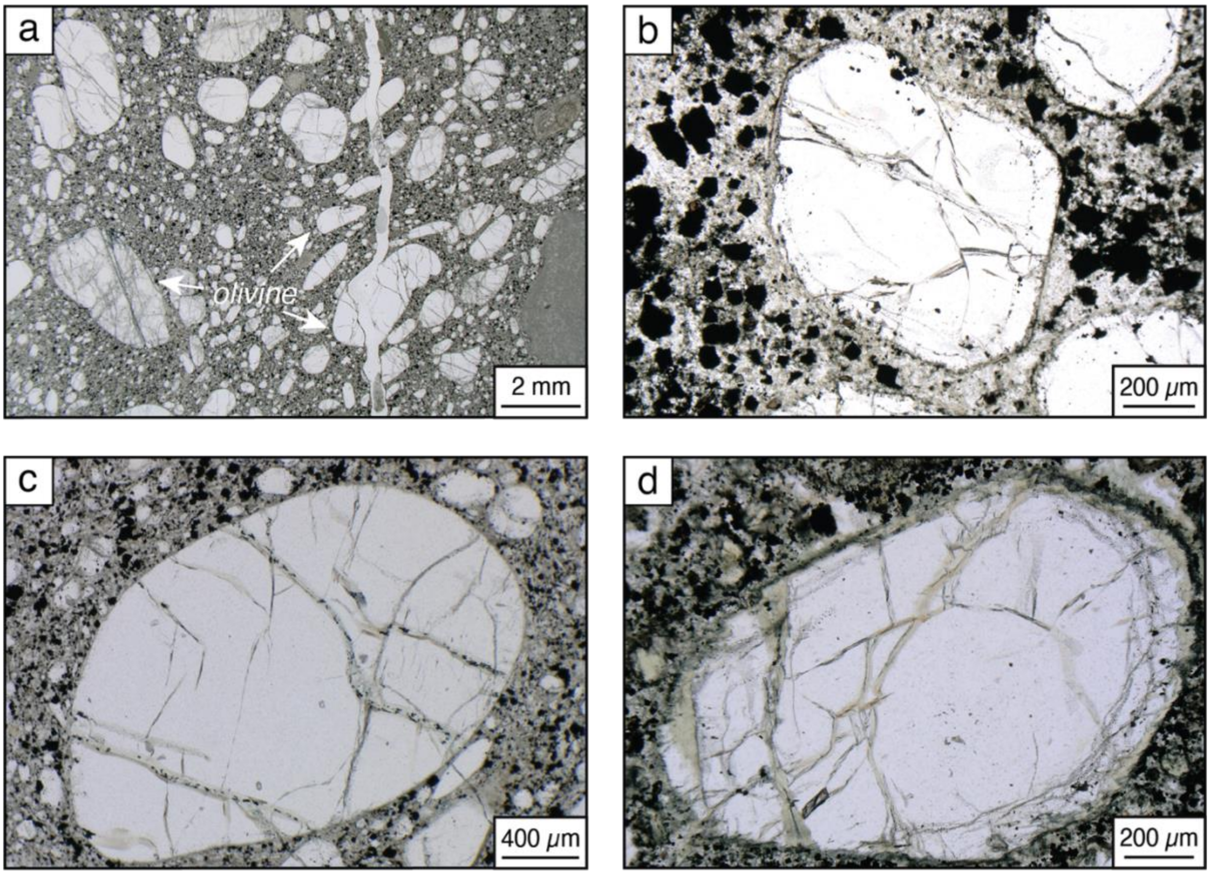

Figure S1. Representative optical images of kimberlites and their olivines with fluid inclusions. a) Thin section photograph of the Internationalnaya kimberlite (Siberia, Russia); note the large amount of colourless olivine. b-d) Optical photographs of olivine macrocrysts from Bultfontein (b), Internationalnaya (c) and Wesselton (d) traversed by fractures and trails of secondary fluid inclusions, and containing primary black inclusions of spinel and/or ilmenite (prominent in panel b).

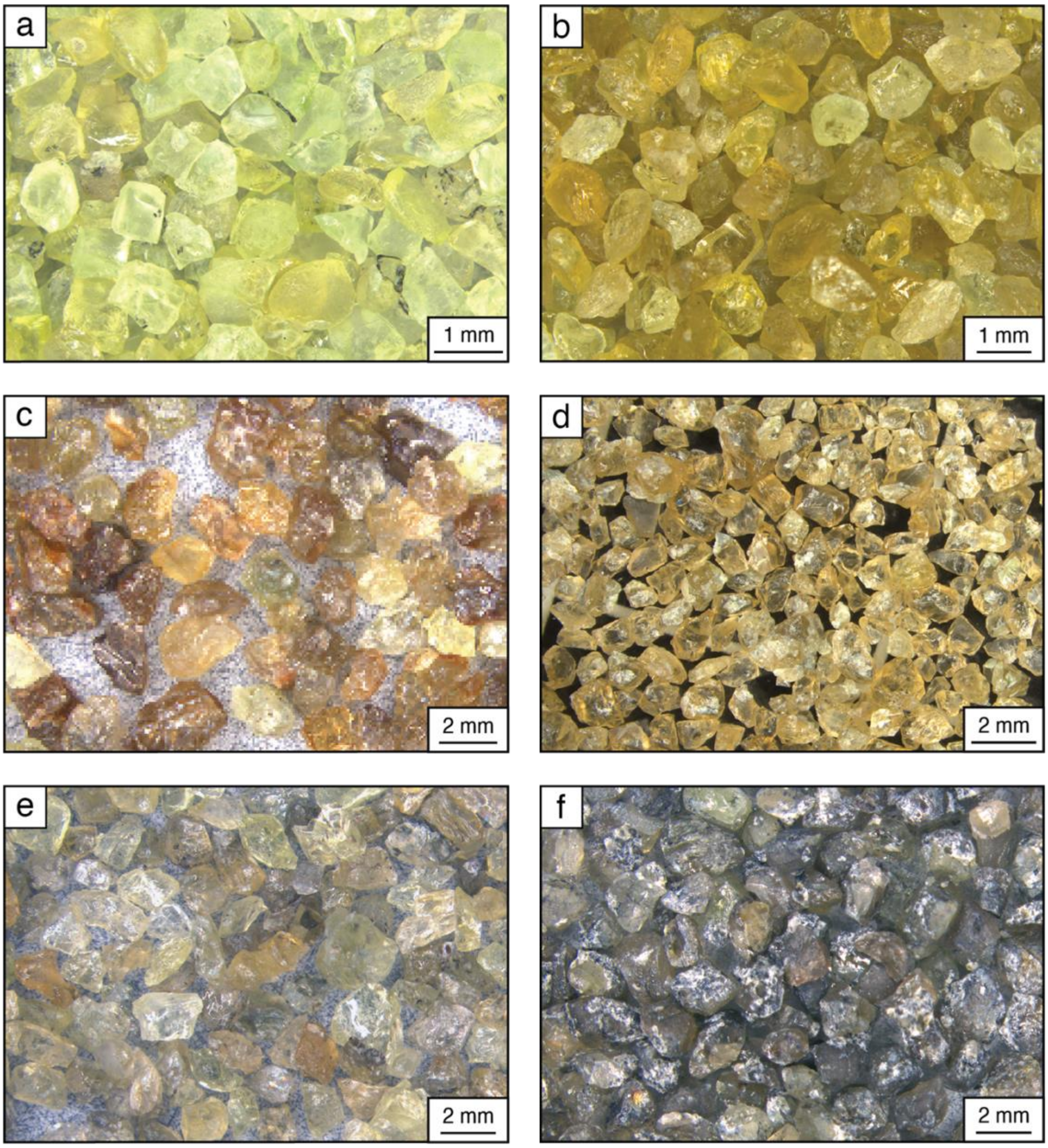

Figure S2. Olivine separates for noble-gas analysis. Optical images of fresh olivines from a) Murowa, b) Victor, c) Karowe, d) Bultfontein, and e) Maniitsoq sample 527 compared to f) a contaminated olivine separate from the same Maniitsoq sample where groundmass material is clearly recognizable attached to most of the olivine grains.

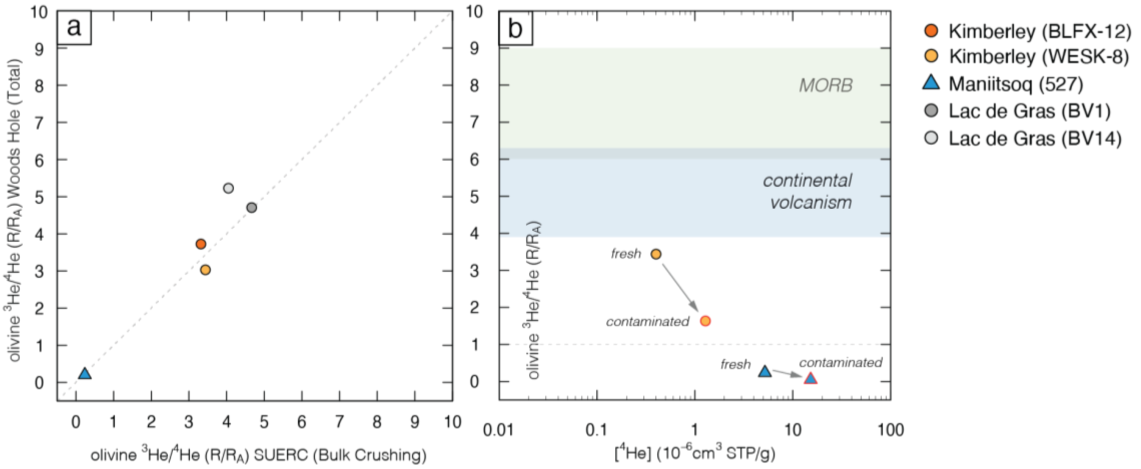

Figure S3. Interlaboratory comparison of He isotope data and effect of groundmass contamination on He isotopes. a) Comparison of $^3He/^4He$ (expressed as ratio R normalised to the atmospheric ratio Ra) measured in olivines from the same kimberlite at Woods Hole (sum of all crushing steps) and SUERC (single crushing step). The dotted line represents the 1:1 correlation. b) $^3He/^4He$ (expressed as ratio R normalised to the atmospheric ratio Ra) *versus* He concentrations of fresh and groundmass-contaminated olivine from the same sample (Wesselton WESK-8 and Maniitsoq 527; data from SUERC). Note the increase in ^4He and decrease in $^3He/^4He$ in the contaminated samples. The $^3He/^4He$ ranges of MORBs unaffected by plume contributions (Kurz and Jenkins, 1981; Graham, 2002) and intracontinental alkaline mafic magmas (Day et al., 2005) are shown for comparison.

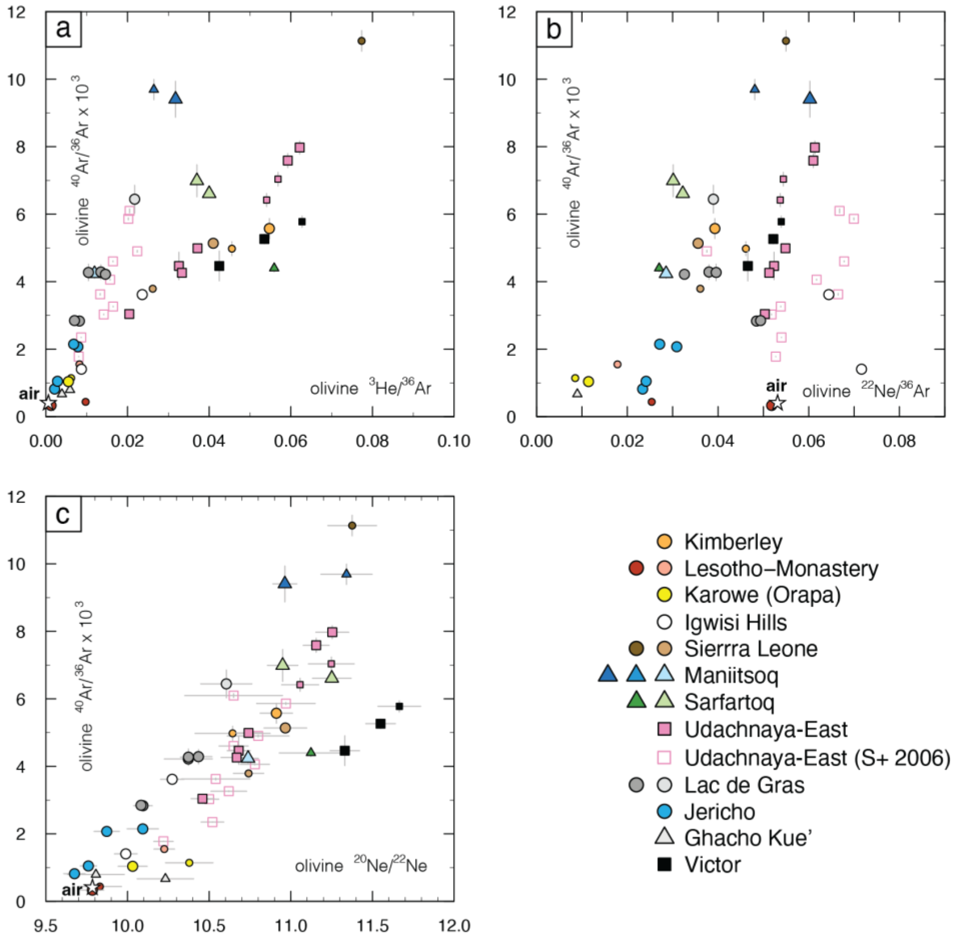

Figure S4. Argon isotope composition of olivine in kimberlites. $^{40}Ar/^{36}Ar$ versus a) $^{3}He/^{36}Ar$, b) $^{22}Ne/^{36}Ar$, and c) $^{20}Ne/^{22}Ne$ in olivine from this study and Sumino et al. (2006; S+ 2006). Each symbol represents analyses of the same sample, with smaller symbols indicating ^{20}Ne counts $<1 \times 10^{-11}$. Error bars represent 1σ uncertainties.

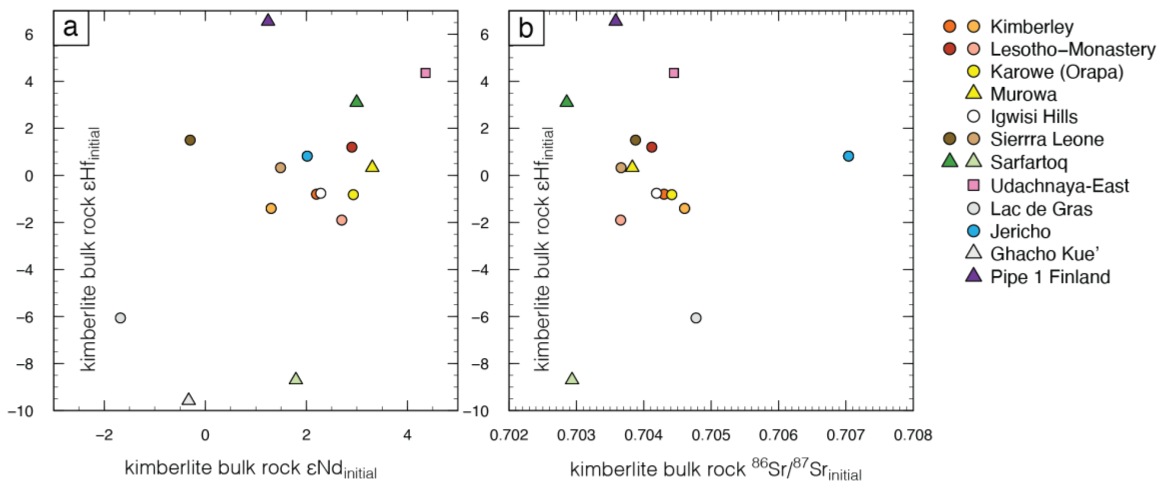

Figure S5. Sr-Nd-Hf isotopes of bulk kimberlite rocks. a) Nd vs Hf isotopes and b) Sr vs Nd isotopes, all corrected for radiogenic ingrowth using kimberlite emplacement ages (Supplementary Data 1), and measured Rb/Sr, Sm/Nd and Lu/Hf ratios (Supplementary Data 3).

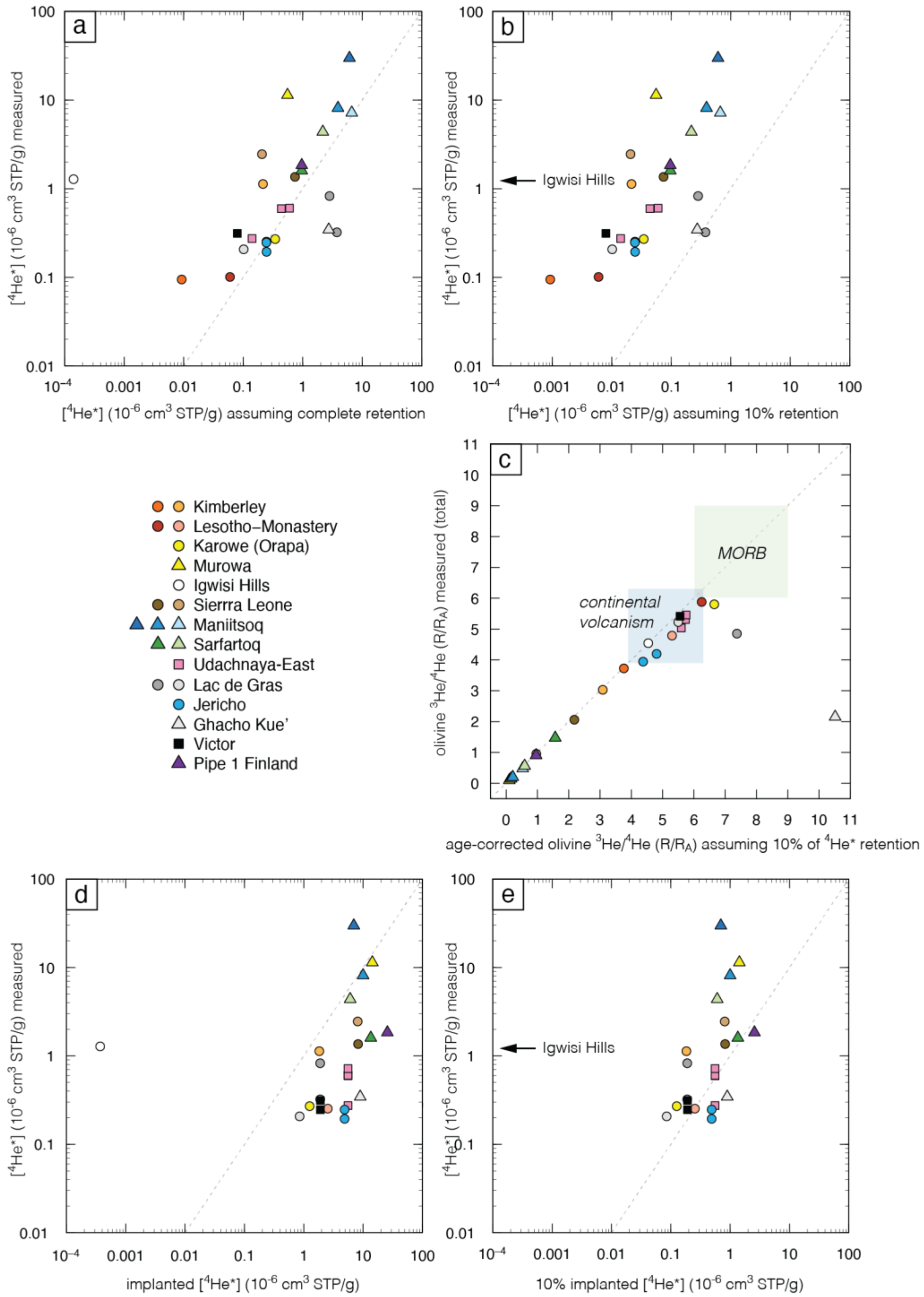

Figure S6. Estimates of radiogenic $^4He^*$ and implanted $^4He^*$ in olivine. a) Measured ^4He (Woods Hole data; sum of all crushing steps) *versus* calculated $^4He^*$ based on measured U-Th contents in dissolved olivine (Supplementary Data 3) and kimberlite emplacement ages (Supplementary Data 1); the dotted line indicates the 1:1 regression. b) Same as panel a, but assuming 10% of $^4He^*$ released by crushing. c) Measured $^3He/^4He$ (Woods Hole data; sum of

all crushing steps) vs age-corrected $^3He/^4He$ assuming 10% of $^4He^*$ retention; the dotted line indicates the 1:1 regression. The $^3He/^4He$ ranges of MORBs unaffected by plume contributions (Kurz and Jenkins, 1981; Graham, 2002) and intracontinental alkaline mafic magmas (Day et al., 2005) are shown for comparison. d) Measured ^4He *versus* implanted $^4He^*$ (I) based on measured U-Th contents in bulk kimberlite rocks (Supplementary Data 3) and kimberlite emplacement ages (Supplementary Data 1); details of the calculations are provided in **Methods**. The dotted line indicates the 1:1 regression. e) Same as panel d, but assuming 10% of implanted $^4He^*$ is released by crushing.

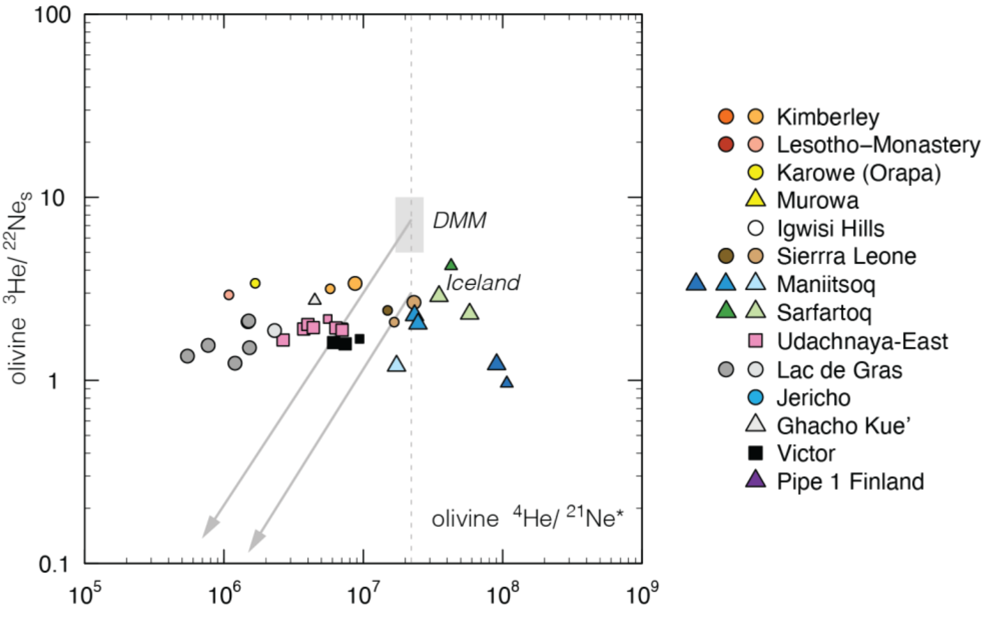

Figure S7. $^4He/^{21}Ne^*$ *versus* $^3He/^{22}Nes$ in olivine from this study. $^{21}Ne^*$ represents nucleogenic Ne that is corrected for air contribution and assuming $^{20}Ne/^{22}Ne = 12.5$ for $^{21}Ne^*$. ^{22}Nes is ^{22}Ne extrapolated to $^{20}Ne/^{22}Ne = 12.5$. Each symbol represents analyses of the same sample, with smaller symbols indicating ^{20}Ne counts $<1 \times 10^{-11}$. The vertical dotted lines represent the mantle (and crustal) production rate of 2.2×10^7 (Yatsevich and Honda, 1997). DMM represent the composition of undegassed MORBs while the arrows reproduce the He loss trends of MORB and Iceland samples (based on the data compiled by Halldorsson et al., 2014).

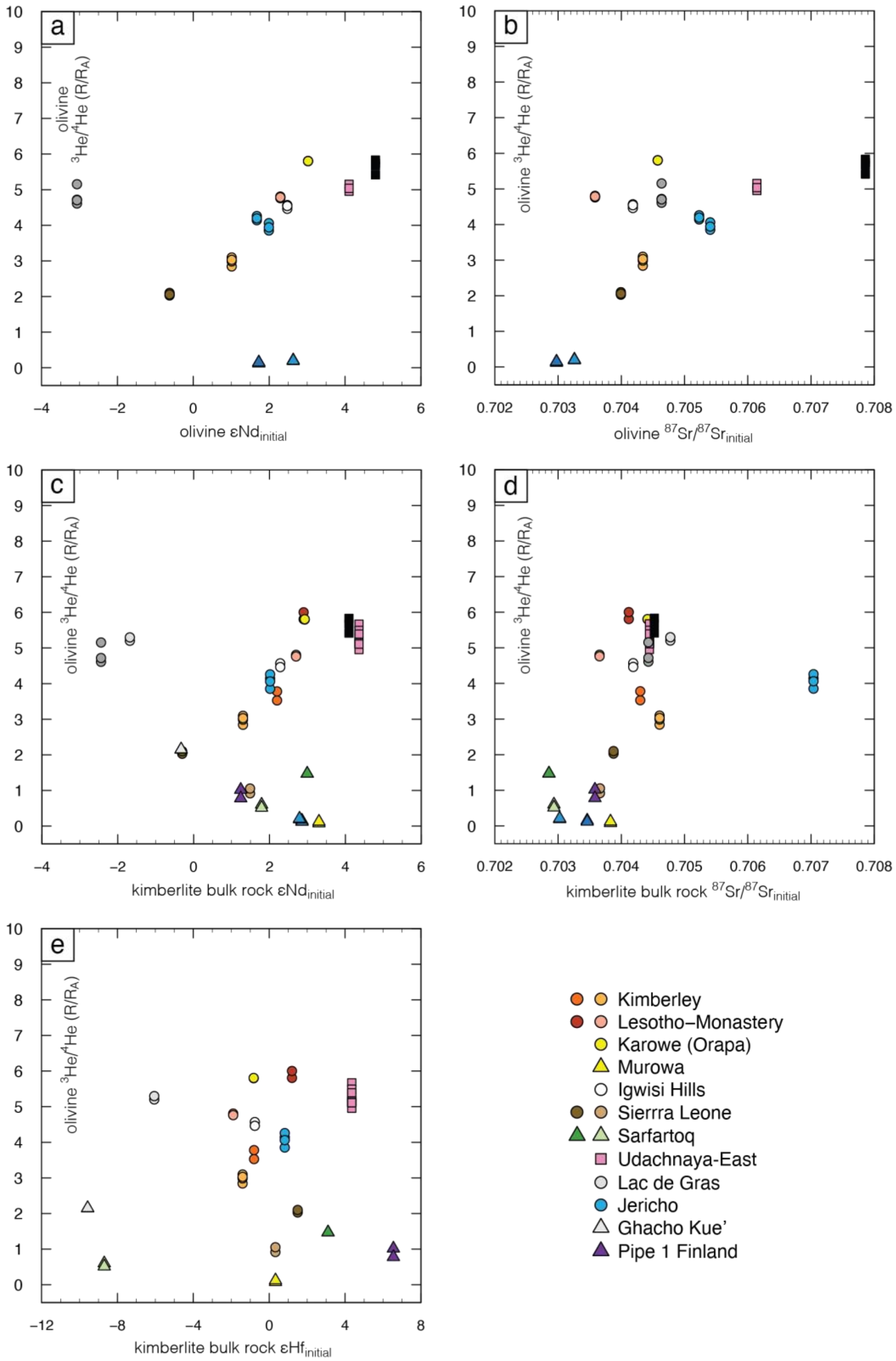

Figure S8. Comparison of olivine He isotopes with olivine and bulk-kimberlite Sr, Nd and Hf isotopes. Olivine $^3He/^4He$ (expressed as ratio R normalised to the atmospheric ratio

Ra; only data acquired in Woods Hole) *versus* olivine a) Nd and b) Sr isotopes, and bulk-kimberlite c) Nd, d) Sr and e) Hf isotopes. For He isotopes, each symbol represents analyses of the same sample. Sr, Nd and Hf isotopes are corrected for radiogenic ingrowth using kimberlite emplacement ages (Supplementary Data 1), and measured Rb/Sr, Sm/Nd and Lu/Hf ratios (Supplementary Data 3).

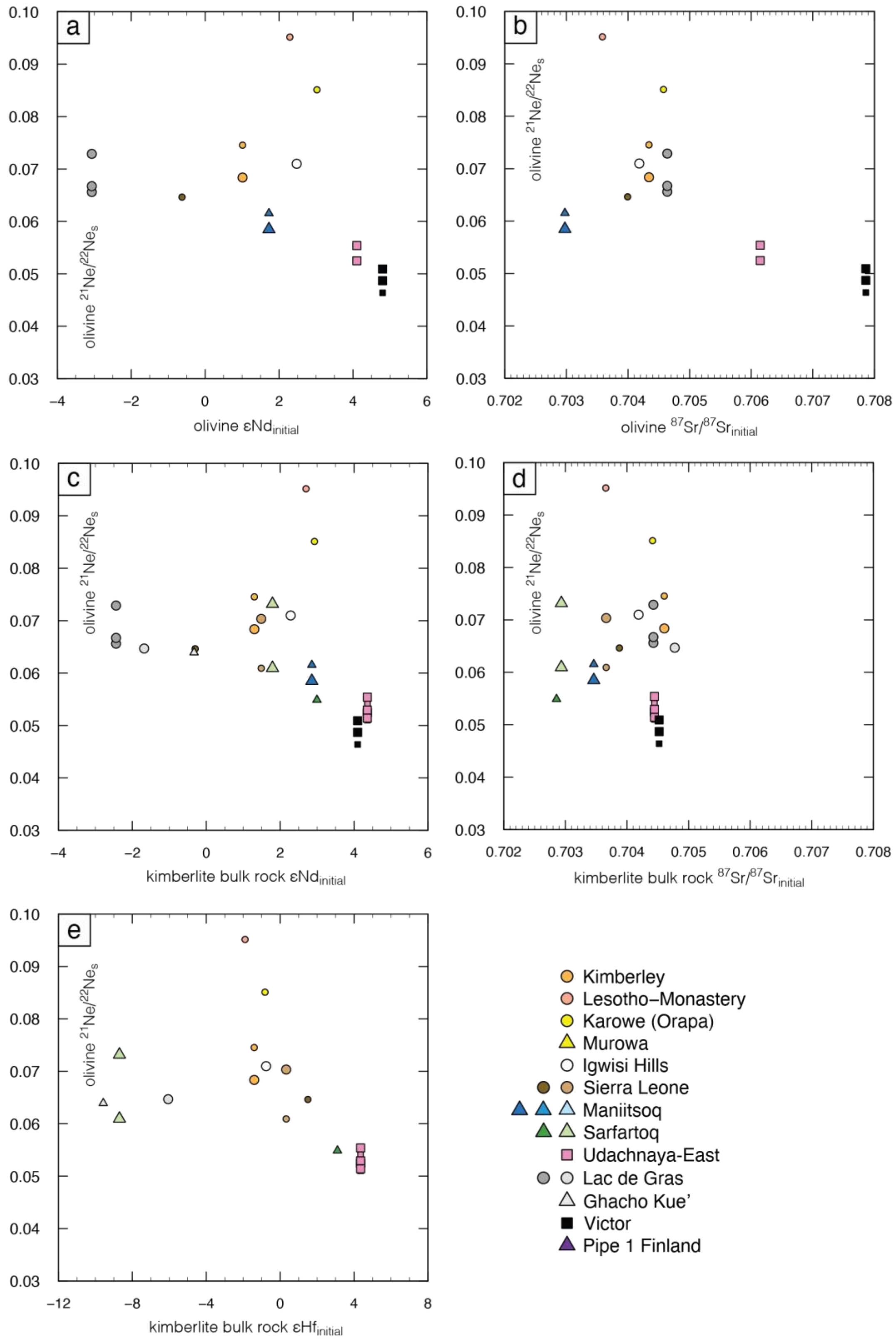

Figure S9. Comparison of olivine Ne isotopes with olivine and bulk-kimberlite Sr, Nd and Hf isotopes. Olivine $^{21}Ne/^{22}Ne_s$ *versus* olivine a) Nd and b) Sr isotopes, and bulk-

kimberlite c) Nd, d) Sr and e) Hf isotopes. For Ne isotopes, each symbol represents analyses of the same sample, with smaller symbols indicating ^{20}Ne counts <1 × 10^{-11}. $^{21}Ne/^{22}Ne_S$ is the extrapolation of $^{21}Ne/^{22}Ne$ to $^{20}Ne/^{22}Ne$ = 12.5. Sr, Nd and Hf isotopes are corrected for radiogenic ingrowth using kimberlite emplacement ages (Supplementary Data 1), and measured Rb/Sr, Sm/Nd and Lu/Hf ratios (Supplementary Data 3).
