# Supplementary material for: Primordial neon and the deep mantle origin of kimberlites
Source: Nat Commun. 2025 Apr 6;16:3281. doi: 10.1038/s41467-025-58625-5 (PMC11972409; doi:10.1038/s41467-025-58625-5)
Supplement: Supplementary file 3 — Description of Additional Supplementary Files [file 41467_2025_58625_MOESM3_ESM.pdf]

Description of Additional Supplementary Files

Supplementary Data 1. List of samples, including analysed material and kimberlite emplacement age.

Supplementary Data 2. He-Ne-Ar concentrations and isotopic compositions for olivine in kimberlites

Supplementary Data 3. Trace element concentrations (ppm) and Sr-Nd-Hf isotope compositions of bulk kimberlite rocks and olivine separates

Supplementary Data 4. Endmember components utilised in He-Ne isotope mixing models
